# Correction to ‘Commodity-specific punishment for experimentally induced defection in cooperatively breeding fish’

**DOI:** 10.1098/rsos.200365

**Published:** 2020-04-08

**Authors:** J. Naef, M. Taborsky

*R. Soc. open sci.* **7**, 191808 (Published Online 12 February 2020) (doi:10.1098/rsos.191808)

This correction refers to an error in the captions of figures 2, 3 and 4. ‘Means’ was used in place of ‘Medians’. This has now been corrected.

